# Nitrite lowers the oxygen cost of ATP supply in cultured skeletal muscle cells by stimulating the rate of glycolytic ATP synthesis

**DOI:** 10.1371/journal.pone.0266905

**Published:** 2022-08-08

**Authors:** Anthony G. Wynne, Charles Affourtit

**Affiliations:** School of Biomedical Sciences, University of Plymouth, Plymouth, United Kingdom; National Institutes of Health, National institute of Diabetes and Digestive and Kidney Diseases, UNITED STATES

## Abstract

Dietary nitrate lowers the oxygen cost of human exercise. This effect has been suggested to result from stimulation of coupling efficiency of skeletal muscle oxidative phosphorylation by reduced nitrate derivatives. In this paper, we report the acute effects of sodium nitrite on the bioenergetic behaviour of cultured rat (L6) myocytes. At odds with improved efficiency of mitochondrial ATP synthesis, extracellular flux analysis reveals that a ½-hour exposure to NaNO_2_ (0.1–5 μM) does not affect mitochondrial coupling efficiency in static myoblasts or in spontaneously contracting myotubes. Unexpectedly, NaNO_2_ stimulates the rate of glycolytic ATP production in both myoblasts and myotubes. Increased ATP supply through glycolysis does not emerge at the expense of oxidative phosphorylation, which means that NaNO_2_ acutely increases the rate of overall myocellular ATP synthesis, significantly so in myoblasts and tending towards significance in contractile myotubes. Notably, NaNO_2_ exposure shifts myocytes to a more glycolytic bioenergetic phenotype. Mitochondrial oxygen consumption does not decrease after NaNO_2_ exposure, and non-mitochondrial respiration tends to drop. When total ATP synthesis rates are expressed in relation to total *cellular* oxygen consumption rates, it thus transpires that NaNO_2_ lowers the oxygen cost of ATP supply in cultured L6 myocytes.

## Introduction

Inorganic nitrate $\left({NO}_{3}^{-}\right)$ is found in beetroot and leafy vegetables such as spinach and rocket [1] and protects against cardiovascular disease [2]. This protection is afforded by nitric oxide formed through reduction of the dietary nitrate [3]. After ingestion, nitrate is first reduced to nitrite by nitrate reductases in bacteria that occupy the posterior part of the tongue [4]. Salivary nitrite is taken up into the circulation lifting the plasma nitrite level up to 600 nM [5]. At this relatively high concentration, and at a low pH and oxygen tension, nitrite can then be reduced to nitric oxide, possibly catalysed by xanthine oxidase [6] or deoxyhaemoglobin [7].

Dietary nitrate has been found to lower the oxygen cost of exercise, as it decreases the respiratory activity required for a set rate of skeletal muscle work [8, 9]. This interesting finding seems at odds with the notion that oxygen uptake for any individual is fixed at a set workload irrespective of age, fitness, diet or pharmacological intervention [10], and has provoked much new research [11]. Meta-analysis of this research confirms that nitrate enhances muscle performance in different exercise contexts [12, 13], but highlights the need for mechanistic understanding. Such understanding may explain why the nitrate benefit does not extend to all types of exercise, why benefit is limited to certain human groups, and why any tested group contains distinct responders and non-responders [14].

The mechanism by which dietary nitrate improves muscle performance remains incompletely understood, but many models predict increased efficiency of myocellular bioenergetics [14]. Increased glycolysis has been ruled out as mechanistic explanation for a decreased oxygen cost of muscle work by the apparent insensitivity of the circulating lactate concentration to nitrate supplementation [8]. A lowered oxygen cost of exercise has instead been attributed to raised efficiency of oxidative ATP supply [15], but this notion is controversial [16, 17]. Dietary nitrate also improves contractile properties of human skeletal muscle [18], which suggests it lowers the ATP cost of work. These mechanisms are not mutually exclusive, as efficiency of oxidative ATP production and ATP consumption may both be increased [19]. It appears widely accepted that the exercise effects of dietary nitrate are mediated by nitric oxide [11], but the evidence is circumstantial. As nitrate/nitrite exposure increases the intracellular level of nitrate, nitrite and nitric oxide in human myocytes [20], any of these species may contribute, in principle, to enhanced muscle function.

Reasoning that nitrate may not be reduced to nitric oxide under the conditions that prevail during the dietary supplementation, and indeed during the submaximal intensity exercise it benefits most, we set out to establish how nitrite, i.e., the reduction intermediate between nitrate and nitric oxide, affects the bioenergetic behaviour of cultured skeletal muscle cells. Calculating rates of intracellular ATP synthesis from real-time cellular oxygen consumption and medium acidification [21], we report here that NaNO_2_ acutely stimulates the rate of glycolytic ATP production in cultured myocytes. This stimulation occurs without negative effect on mitochondrial ATP synthesis, and thus lowers the oxygen cost of total ATP supply.

## Material and methods

### Tissue culture

Clonal rat myoblasts (L6.C11) were obtained from the European Collection of Cell Culture. These L6 myoblasts were seeded 24 h before experiments at 30,000 cells per well on either XF24 tissue culture plates (Agilent-Seahorse) for extracellular flux analysis, or on 96-well plates (Corning) for glucose uptake assays. Cells were studied between passages 11 and 20 and were grown in DMEM supplemented with 5 mM glucose, 10% (v/v) fetal bovine serum under a humidified carbogen atmosphere of 5% CO_2_ and 95% air at 37°C. To allow cell differentiation, myoblasts were cultured to complete confluence in fully supplemented DMEM on XF24 or 96-well plates, at which point the serum concentration was decreased to 2% (v/v). When cells were cultured in this ‘light’-serum medium for 14 days–i.e., longer than the 8–10 days we stuck to before [22]–and growth medium was refreshed every 2–3 days, the L6 myoblasts differentiated into myotubes that contract spontaneously.

### Cellular bioenergetics

Oxygen consumption and medium acidification by myoblasts and spontaneously contracting myotubes were measured with a Seahorse XF24 extracellular flux analyser (Agilent). Before the assay, cells were washed into a Hepes-buffered Krebs-Ringer medium (KRH) comprised of 135 mM NaCl, 3.6 mM KCl, 2 mM Hepes (pH 7.4), 0.5 mM MgCl_2_, 1.5 mM CaCl_2_, 0.5 mM NaH_2_PO_4_, and 5 mM glucose. Cells were allowed to equilibrate in this medium for 30 min under air at 37°C, and were then transferred to the XF24 analyser for another 12-min equilibration. Oxygen consumption (OCR) and extracellular acidification (ECAR) rates were recorded 3x (measurement cycle: 2-min mix, 2-min wait and 3-min measure) at which point 0.1–5 μM NaNO_2_ was added; KRH and NaNO_3_ (up to 500 μM) were used as controls. Following about ½-h (26 min) incubation, 5 μg/mL oligomycin, 1 μM carbonyl cyanide-p-trifluoromethoxyphenylhydrazone (FCCP) or N5,N6-bis(2-fluorophenyl)[1,2,5]oxadiazolo[3,4-b]pyrazine-5,6-diamine (BAM15), and 1 μM rotenone with 1 μM antimycin A were added sequentially to inhibit the ATP synthase, uncouple oxidative phosphorylation, and inhibit the mitochondrial electron transfer chain, respectively. OCRs (calculated from time-resolved oxygen concentrations applying the *Akos* algorithm [23]) and ECARs were normalised to the number of nuclei quantified by 4’,6-diamidino-2-phenylindole (DAPI) staining as described before [24]. It was thus determined that each well contained on average 27K myoblast nuclei–i.e., within experimental variation of the 30K/well seeding density–or 39K myotube nuclei. OCRs resistant to rotenone and antimycin A were subtracted from all other OCRs to correct for non-mitochondrial oxygen uptake. Mitochondrial respiration coupled to ATP synthesis or associated with proton leak was gauged from the oligomycin-sensitive and oligomycin-resistant OCRs, respectively, while mitochondrial respiratory capacity was estimated from uncoupled respiration. Coupling efficiency of oxidative phosphorylation was defined as the percentage of basal respiration used to make ATP, and cell respiratory control as the ratio between uncoupled and oligomycin-inhibited respiration [25].

The rates of glycolytic and mitochondrial ATP synthesis were derived from cellular oxygen uptake and medium acidification as described by Mookerjee *et al*. [21], assuming myocyte energy metabolism was fuelled exclusively by glucose. The mitochondrial ATP supply rate was calculated from mitochondrial respiration coupled to ATP synthesis using a P/O ratio for glucose-fuelled TCA cycle turnover of 0.12 and a P/O ratio for glucose-fuelled oxidative phosphorylation of 2.50 [21], thus assuming that glycolytic reducing power is transferred to mitochondria by the malate-aspartate shuttle alone. ATP-synthesis-coupled respiration was approximated from the oligomycin-sensitive oxygen consumption after correcting for the small underestimation owing to oligomycin-induced hyperpolarisation of the mitochondrial inner membrane [21]. Glycolytic ATP synthesis was defined as the net ATP produced during glucose breakdown to pyruvate irrespective of pyruvate’s destiny. The glycolytic ATP supply rate was thus calculated from medium acidification to account for pyruvate that was reduced to lactate^−^and H^+^ (ATP:lactate = 1:1) and from mitochondrial respiration to account for the pyruvate that was oxidised to bicarbonate (ATP:O_2_ = 0.33:1). A KRH buffering power of 0.681 mpH x μM^-1^ H^+^ was used to convert the ECAR to a proton production rate (PPR) assuming an effective XF24 measurement volume of 22.7 μL [25]. The PPR was corrected for medium acidification accounted for by bicarbonate^−^plus H^+^ that emerges from pyruvate oxidation to reflect contribution of lactate^−^and H^+^ only [26]. Lactate production rates were also measured directly by lactate dehydrogenase assay of medium samples taken after completion of the XF run, and these rates were normalised to total protein [26].

### Glucose uptake

2-Deoxyglucose uptake by myoblasts and myotubes was measured as described previously [22]. Cells were assayed and washed in glucose-free KRH (see previous section), and were lysed in buffer containing 0.1 N HCl and 0.1% (w/v) Triton X100 at room temperature. This acidic lysis method offered a higher signal-to-noise ratio than the high-temperature alkaline procedure we used before [22].

### Statistical analysis

Differences between myocellular differentiation state and bioenenergetic condition, as well as NaNO_2_ and NaNO_3_ effects on a series of bioenergetic parameters, were evaluated for statistical significance using GraphPad Prism (version 9 for Windows). Data are presented as means ± SEM of a number of wells that were assayed once each, and that were sampled from multiple plates. Well measurement and assay plate numbers, as well as the nature of the statistical tests, are specified in the figure legends.

## Results

### Contractile myotubes rely more strongly on glycolytic ATP supply than static myoblasts

**Fig 1:**
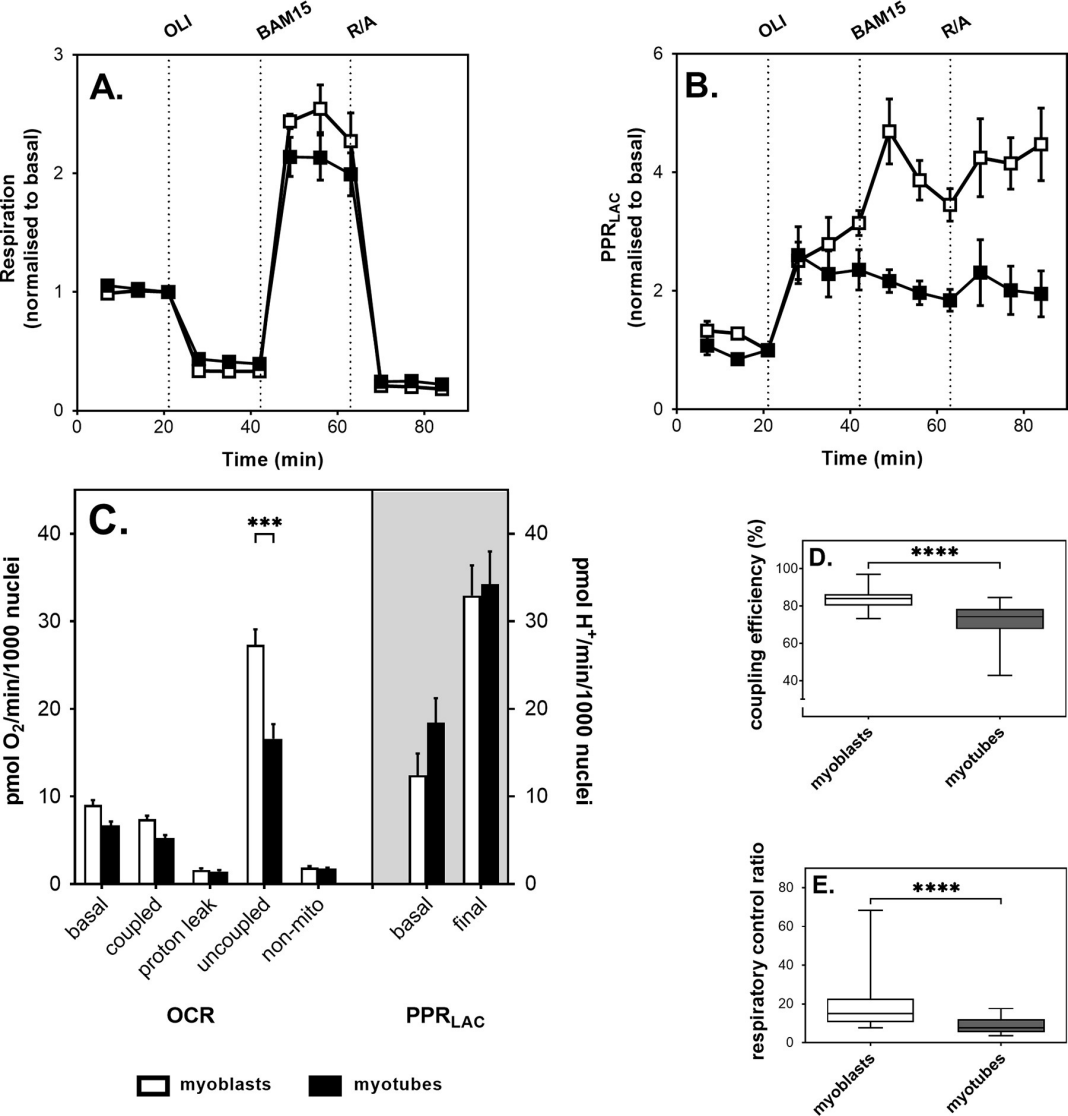
Myocellular respiration and proton release. Oxygen uptake and medium acidification by static L6 myoblasts (open symbols) and spontaneously contracting myotubes (filled symbols) were measured by extracellular flux (XF) analysis. Panels **A** and **B**: Respiratory and medium acidification traces, respectively, are based on the means ± SEM of 4 individual extracellular flux runs with the differentiation states measured 3–5 times in each. Medium acidification traces were corrected for CO_2_ contribution and thus reflect the proton production rate due to release of lactate^−^and H^+^ (PPR_LAC_) alone. Rates were normalised to the 3^rd^ measurement and were obtained in the absence of any respiratory effector or in the cumulative presence of 5 μg/mL oligomycin (OLI), 1 μM uncoupler (BAM15), and a mixture of 1 μM rotenone and 1 μM antimycin A (R/A). Panel **C**: After normalisation to the number of myocyte nuclei, respiratory and proton production rates were used to calculate the basal oxygen consumption and medium acidification rates as well as the oxygen uptake activity that was coupled to ATP synthesis (coupled) or associated with mitochondrial proton leak. The maximum mitochondrial respiratory rate (uncoupled) and the rate of non-mitochondrial oxygen consumption (non-mito) are shown too, as is the PPR_LAC_ measured after addition of all effectors (final). Data are means ± SEM of 13–14 well measurements sampled from 4 independent XF assays. Extracellular flux differences between myoblasts and myotubes were evaluated for statistical significance by 2-way ANOVA applying a Šídák’s multiple comparisons test (*** P < 0.001). Panels **D** and **E**: Coupling efficiencies and cell respiratory control ratios, respectively, were calculated from the data shown in Panel **C** and the control (0 μM NaNO_2_) data shown in Fig 4. Box-and-whiskers plots represent 25–34 measurements sampled from 8–9 independent XF assays. Statistical significance of the differences between myoblasts and myotubes (**** P < 0.0001) was evaluated by Mann Whitney tests.

Prolonged growth of L6 myocytes in low-serum medium leads to formation of spontaneously contracting myotubes (https://www.youtube.com/watch?v=w5UU5UiEi3Q). We subjected these contractile cells to extracellular flux analysis to compare their bioenergetic behaviour with that of resting myoblasts. In both differentiation states, the basal OCR decreases when mitochondrial ATP synthesis is inhibited by oligomycin. Mitochondrial uncoupling stimulates the OCR, a little more strongly in myoblasts than in myotubes, and inhibition of mitochondrial electron transfer diminishes it (Fig 1A). Myocytes compensate for their inability to make ATP through oxidative phosphorylation by raising their rate of anaerobic glycolysis in response to oligomycin, as is clear from the increased proton production rate due to release of lactate^−^and H^+^ (PPR_LAC_, Fig 1B). Normalising OCR and PPR_LAC_ to number of myocyte nuclei allows statistical evaluation of differences in oxygen consumption and lactate-induced medium acidification between myoblasts and myotubes (Fig 1C). Basal, ATP-synthesis-coupled and uncoupled respiration are all lower in myotubes than in myoblasts, but only the uncoupled rate difference is statistically significant. Neither respiration associated with proton leak nor *non*-mitochondrial oxygen consumption differs between differentiation states. Lactate-related medium acidification is somewhat higher in myotubes than myoblasts, particularly in the absence of respiratory effectors, but not significantly so (Fig 1C). The proportion of myocyte respiration that is used to make ATP (Fig 1D) is lower in myotubes (74%) than in myoblasts (84%). This difference in coupling efficiency of oxidative phosphorylation is statistically significant and might be due to the higher workload faced by spontaneously contracting myotubes than by resting myoblasts, much akin to the comparably low fuel efficiency of car engines running at high speed. Indeed, low myotube coupling efficiency resembles the low value reported for intact perfused rat muscle [27]. Consistently, cellular respiratory control, i.e., the ratio of uncoupled and oligomycin-inhibited respiration, is lower in myotubes than myoblasts (Fig 1E).

**Fig 2:**
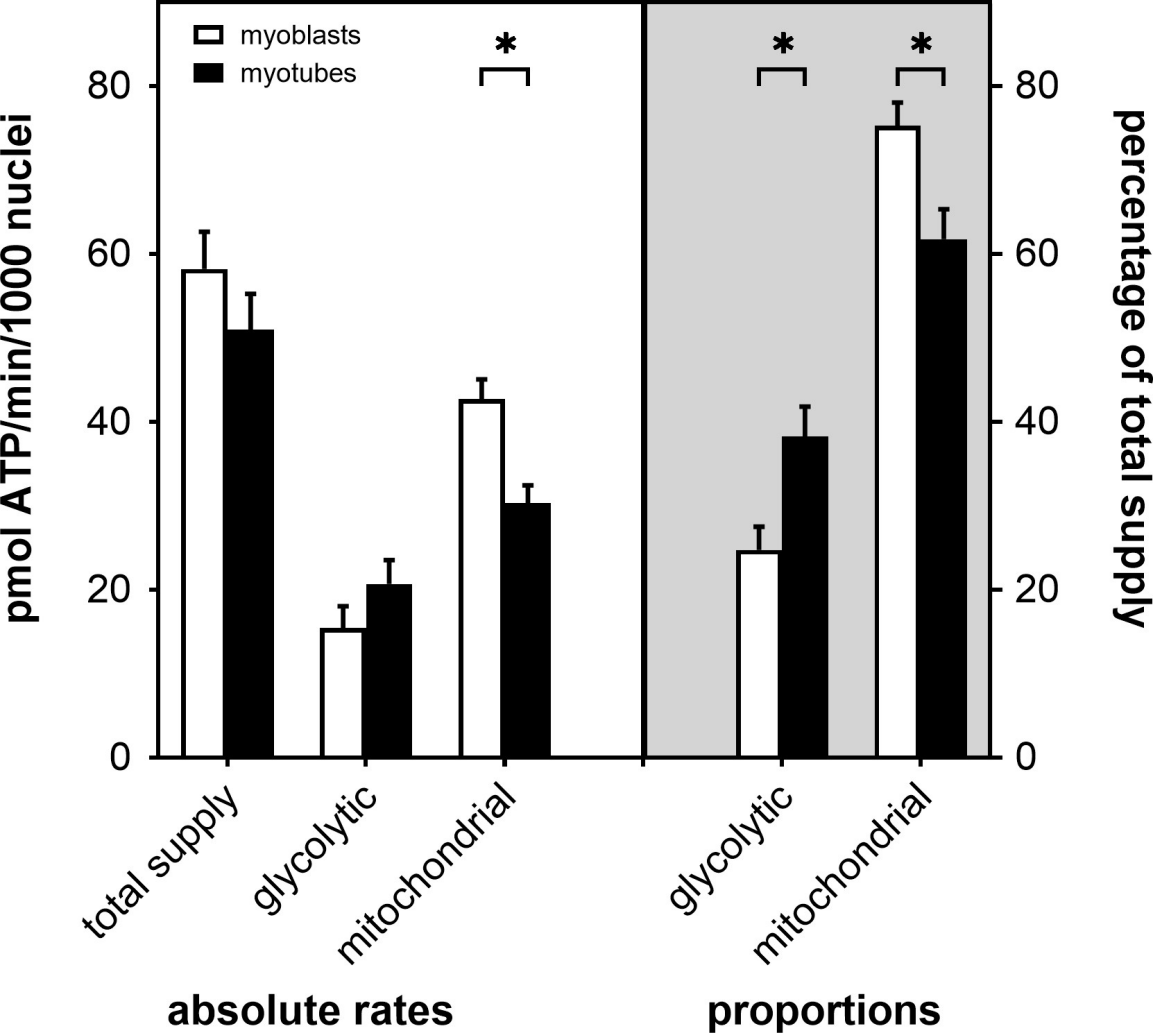
Myocellular ATP supply. Rates of glycolytic and mitochondrial ATP synthesis were calculated from data shown in Fig 1C and were normalised to number of myocyte nuclei (absolute rates) or expressed as percentage of the combined, i.e., total ATP supply (proportions). Myoblast (open bars) and myotube (filled bars) data are means ± SEM of 13–14 well measurements sampled from 4 separate XF assays. Bioenergetic differences between myoblasts and myotubes were evaluated for statistical significance by 2-way ANOVA applying a Šídák’s multiple comparisons test (* P < 0.05).

From basal and oligomycin-sensitive OCR and basal PPR_LAC_ we then calculated absolute ATP supply rates, and found that total supply flux is marginally, but not statistically significantly, higher in myoblasts than in myotubes (Fig 2). Mitochondrial ATP supply is also faster in myoblasts than myotubes, while glycolytic ATP supply tends to be slower (Fig 2). When normalised to total ATP supply, the rate differences are statistically significant, which shows that L6 myocytes become more glycolytic after differentiation to contractile myotubes.

### Nitrite does not affect coupling efficiency of oxidative phosphorylation

**Fig 3:**
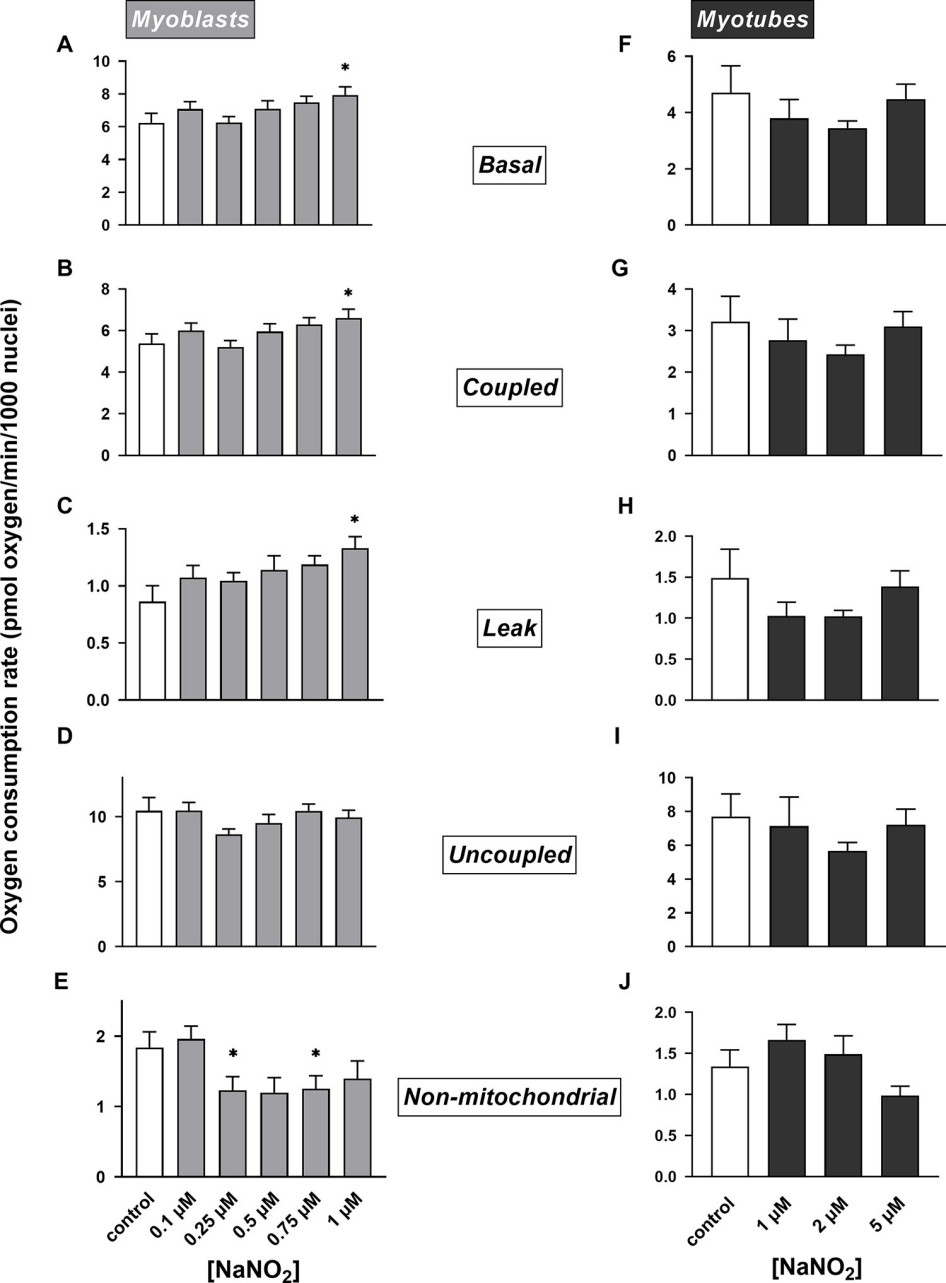
Nitrite effects on myocellular respiration. Mitochondrial and non-mitochondrial oxygen uptake rates were measured in static myoblasts and spontaneously contracting myotubes after a ½-hour exposure to NaNO_2_ as described in Materials and Methods. Respiratory activities were normalised to number of myocyte nuclei and were obtained in the absence of effectors (Basal) or in the cumulative presence of 5 μg/mL oligomycin (Leak), 1 μM FCCP (Uncoupled) and a mix of 1 μM rotenone and 1 μM antimycin A (Non-mitochondrial). Oligomycin-sensitive oxygen uptake was used to estimate respiration coupled to ATP synthesis (Coupled). Data are means ± SEM of 12–20 well measurements sampled from 4–5 independent XF assays. NaNO_2_ effects were evaluated for statistical significance by combined Kruskal-Wallis and Dunn’s tests. Respiratory rates labelled with an asterisk differ significantly (P < 0.05) from the relevant (0 μM NaNO_2_) control rate.

Seeking to establish how the bioenergetics of skeletal muscle cells are affected by nitrite, we exposed resting L6 myoblasts and spontaneously contracting myotubes to NaNO_2_ during the extracellular flux assays at concentrations within one order of magnitude of the circulating nitrite level seen after dietary nitrate supplementation [5]. Given the reported stimulation of oxidative ATP synthesis efficiency by dietary nitrate [15], we first measured possible NaNO_2_ effects on myocyte respiration (Fig 3). Our data show that a ½-hour NaNO_2_ exposure is sufficient to raise the basal oxygen consumption of myoblasts (Fig 3A). This NaNO_2_-induced respiratory rise is statistically significant at 1 μM and is due to stimulation of respiration linked to both ATP synthesis (Fig 3B) and mitochondrial proton leak (Fig 3C). NaNO_2_ does not affect uncoupled respiration (Fig 3D) and lowers non-mitochondrial oxygen consumption, although the effect is not statistically significant at every nitrite concentration (Fig 3E). NaNO_2_ effects on respiration are not seen in myotubes, even when applied up to 5 instead of 1 μM (Fig 3F–3J).

**Fig 4:**
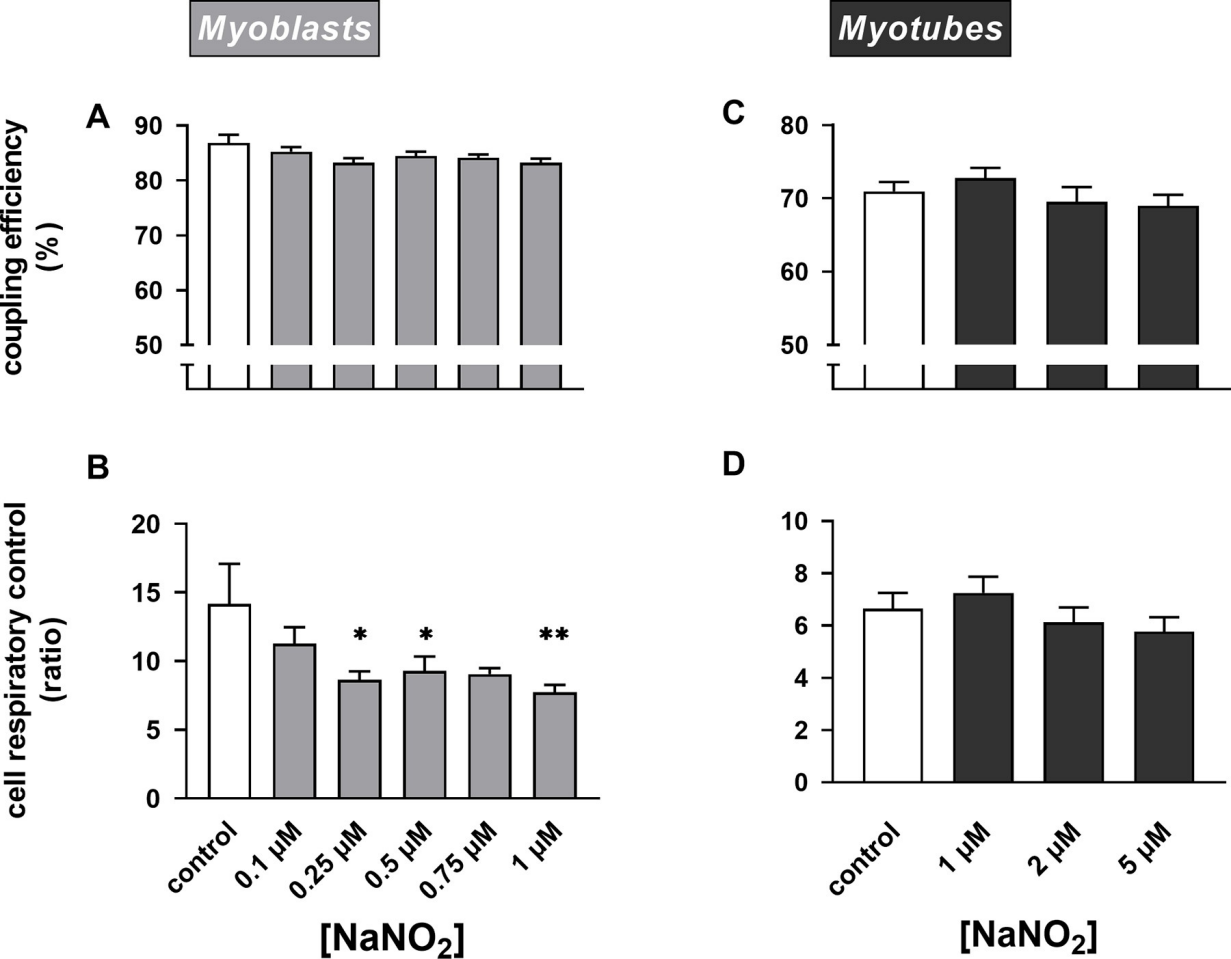
Nitrite lowers mitochondrial efficiency. Respiratory rates shown in Fig 3 were used to calculate coupling efficiency of oxidative phosphorylation (Panels **A** and **C**) and cell respiratory control ratios (Panels **B** and **D**) in static myoblasts and spontaneously contracting myotubes. Data are means ± SEM of 12–20 well measurements sampled from 4–5 independent XF assays. NaNO_2_ effects were evaluated for statistical significance by combined Kruskal-Wallis and Dunn’s tests. Parameters labelled with asterisks differ significantly (* P < 0.05 and ** P < 0.01) from the relevant (0 μM NaNO_2_) control rate.

As NaNO_2_ stimulates both respiration linked to proton leak and respiration coupled to ATP synthesis in myoblasts, it leaves coupling efficiency of oxidative phosphorylation unaffected (Fig 4A). The increased proton leak is responsible for the decreased cell respiratory control ratio (Fig 4B). Consistent with a lack of respiratory effects, nitrite does not affect coupling efficiency (Fig 4C) or cell respiratory control (Fig 4D) in myotubes. Notably, the lack of NaNO_2_ effect on mitochondrial coupling efficiency is at odds with the reported stimulatory effect of NaNO_3_ on the P/O ratio of skeletal muscle mitochondria [15], but is consistent with the more recently reported lack of beneficial effect of dietary nitrate on mitochondrial efficiency [16, 17].

### Nitrite increases the rate of glycolytic and total ATP synthesis

**Fig 5:**
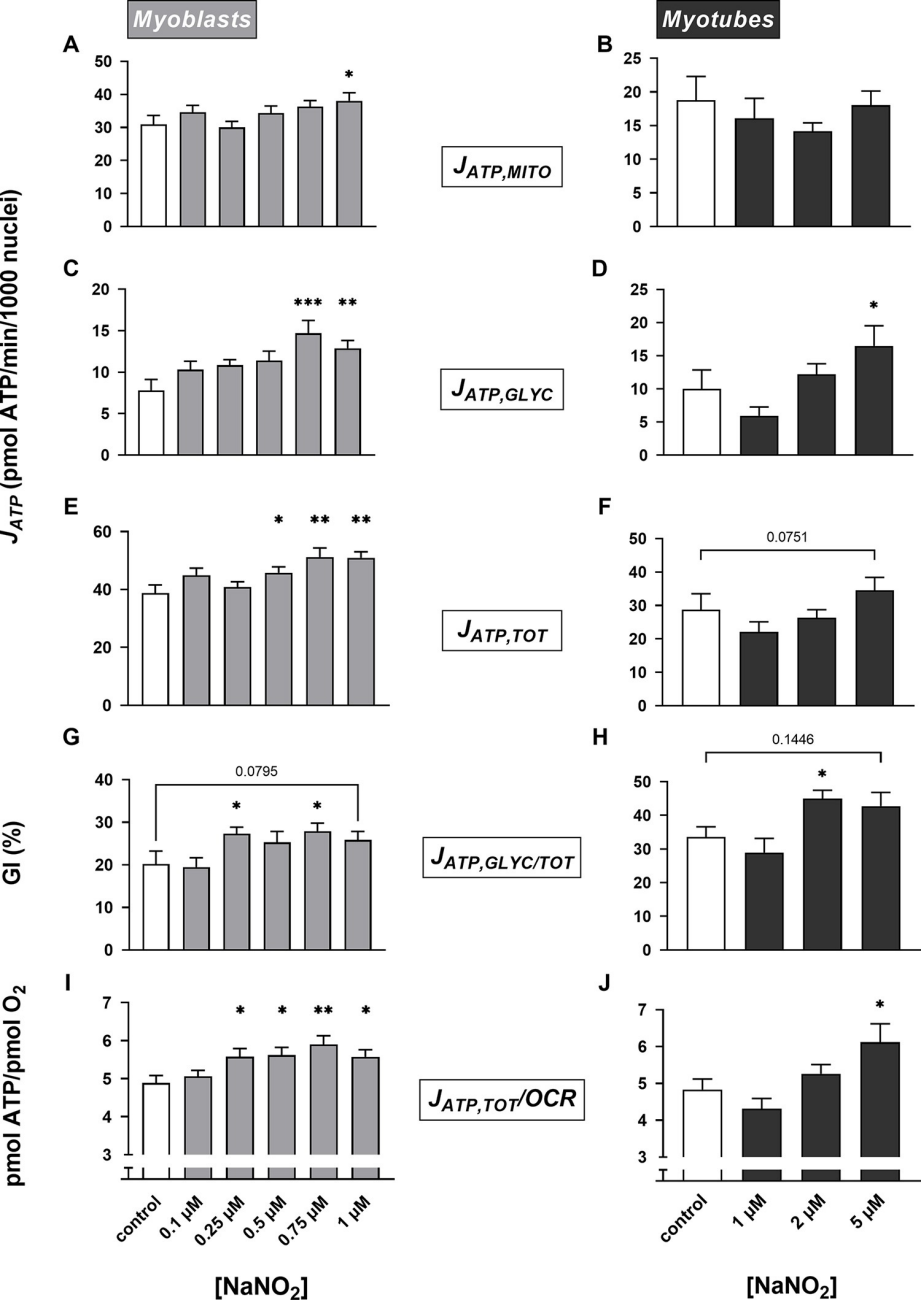
Nitrite effects on ATP supply. Respiratory rates shown in Fig 3 were used, combined with concomitantly measured acidification rates (*cf*. Fig 1B and 1C), to calculate rates of mitochondrial (Panels **A** and **B**), glycolytic (Panels **C** and **D**) and total (Panels **E** and **F**) ATP supply (J_ATP,MITO_, J_ATP,GLYC_ and J_ATP,TOT_, respectively) in static myoblasts and spontaneously contracting myotubes. Glycolytic ATP synthesis rates are also shown as percentages of total ATP supply rate (J_ATP,GLYC/TOT_, Panels **G** and **H**), i.e., as myocellular glycolytic indices (GI). Furthermore, total ATP synthesis rates are normalised to the total cellular oxygen consumption rate (J_ATP,TOT_/OCR, Panels **I** and **J**). Data are means ± SEM of 12–21 well measurements sampled from 4–5 independent XF assays. NaNO_2_ effects were evaluated for statistical significance by combined Kruskal-Wallis and Dunn’s tests. Parameters labelled with asterisks differ significantly (* P < 0.05, ** P < 0.01, *** P < 0.001) from the relevant (0 μM NaNO_2_) control rate.

**Fig 6:**
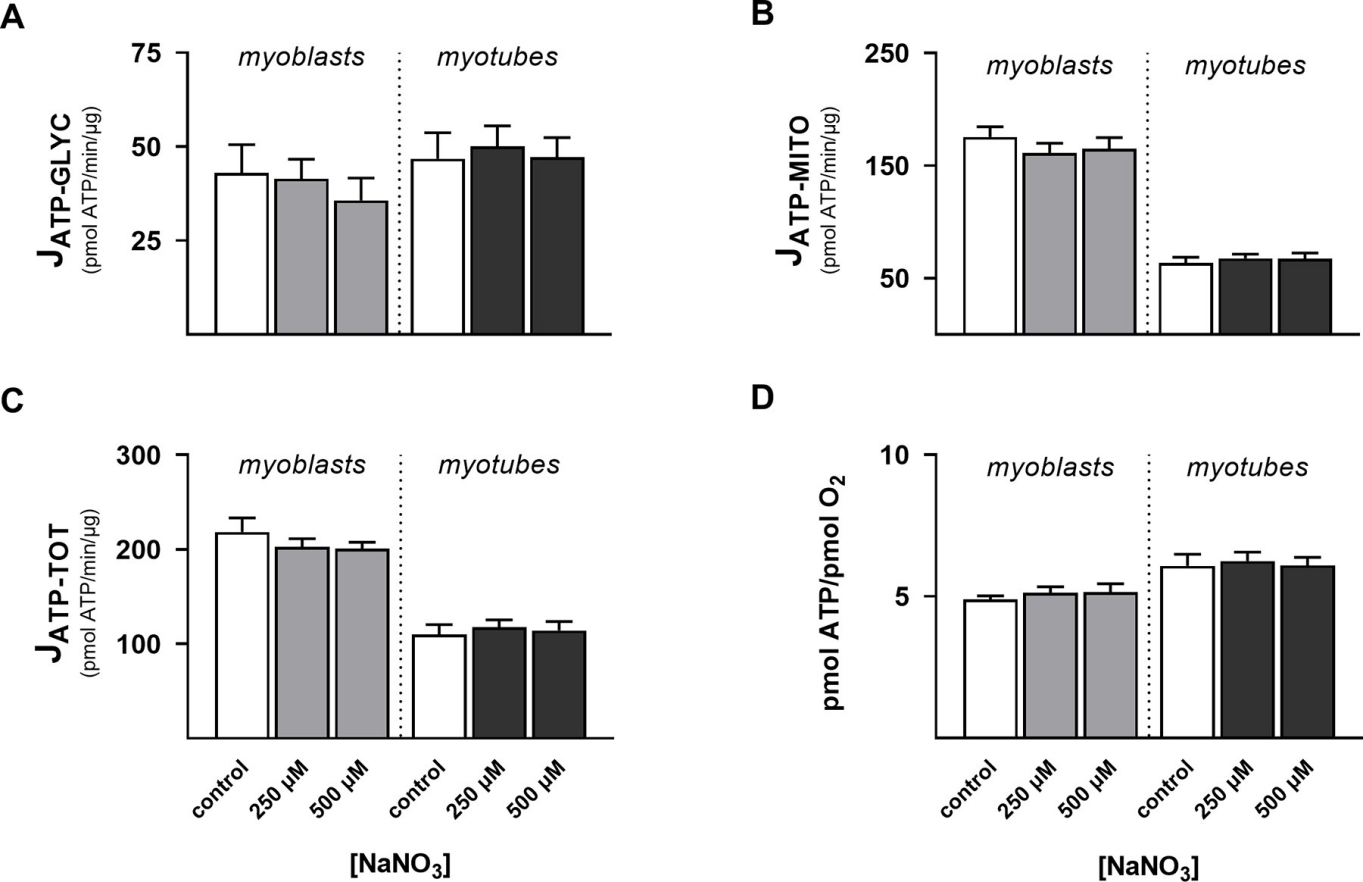
Nitrate effects on ATP supply. Rates of mitochondrial, glycolytic and total ATP supply (J_ATP,MITO_, J_ATP,GLYC_ and J_ATP,TOT_, respectively) in static myoblasts and spontaneously contracting myotubes were calculated from oxygen uptake and medium acidification data obtained after a ½-hour exposure to NaNO_3_. Data are means ± SEM of 13–18 well measurements sampled from 4 independent XF assays. NaNO_3_ effects were evaluated for statistical significance by combined Kruskal-Wallis and Dunn’s tests and were found non-significant.

Probing the lack of nitrite effect on mitochondrial coupling efficiency further, we next assessed if NaNO_2_ affected the *rate* of oxidative phosphorylation. It transpires that NaNO_2_ increases the rate of mitochondrial ATP synthesis in myoblasts, but only significantly so at 1 μM (Fig 5A), and has no significant effect in myotubes (Fig 5B). Interestingly, calculating ATP supply flux from the XF data [21] revealed that NaNO_2_ raises the rate of glycolytic ATP supply both in myoblasts, at 0.75 and 1 μM (Fig 5C), and in myotubes, at 5 μM (Fig 5D). Consistent with this surprising discovery, enzyme-based measurement of lactic acid release by myotubes during extracellular flux assays shows that NaNO_2_ increases the specific lactate production rate from 1.3 ± 0.52 to 3.1 ± 1.3 pmol/min/μg protein (P < 0.05). NaNO_2_ stimulation of glycolytic ATP supply flux is evident in myoblasts and myotubes, either when rates are normalised to number of nuclei (Fig 5C and 5D) or to the overall ATP supply flux (Fig 5G and 5H). The total ATP supply is itself increased by NaNO_2_, an acute effect that is significant in myoblasts at 0.5, 0.75 and 1 μM (Fig 5E) and tends to significance in myotubes at 5 μM (Fig 5F). Notably, NaNO_2_ has a statistically significant stimulatory effect on total ATP supply in myoblasts, at 0.25, 0.5, 0.75 and 1 μM (Fig 5I), and myotubes, at 5 μM (Fig 5J), when this ATP supply is expressed in relation to basal cellular respiration. The increased ATP/O_2_ ratio (Fig 5I and 5J) thus indicates that NaNO_2_ lowers the apparent oxygen cost of total ATP synthesis. Effects on myocellular ATP supply are specific to nitrite, as *nitrate* does not affect the rates of glycolytic (Fig 6A), mitochondrial (Fig 6B) or total (Fig 6C) ATP supply in myoblasts or myotubes that were exposed for a ½ hour to NaNO_3_ at concentrations seen in circulation after dietary nitrate supplementation [5]. Notably, nitrate leaves the oxygen cost of ATP supply unaffected as well (Fig 6D).

### Nitrite increases the cellular glycolytic index

**Fig 7:**
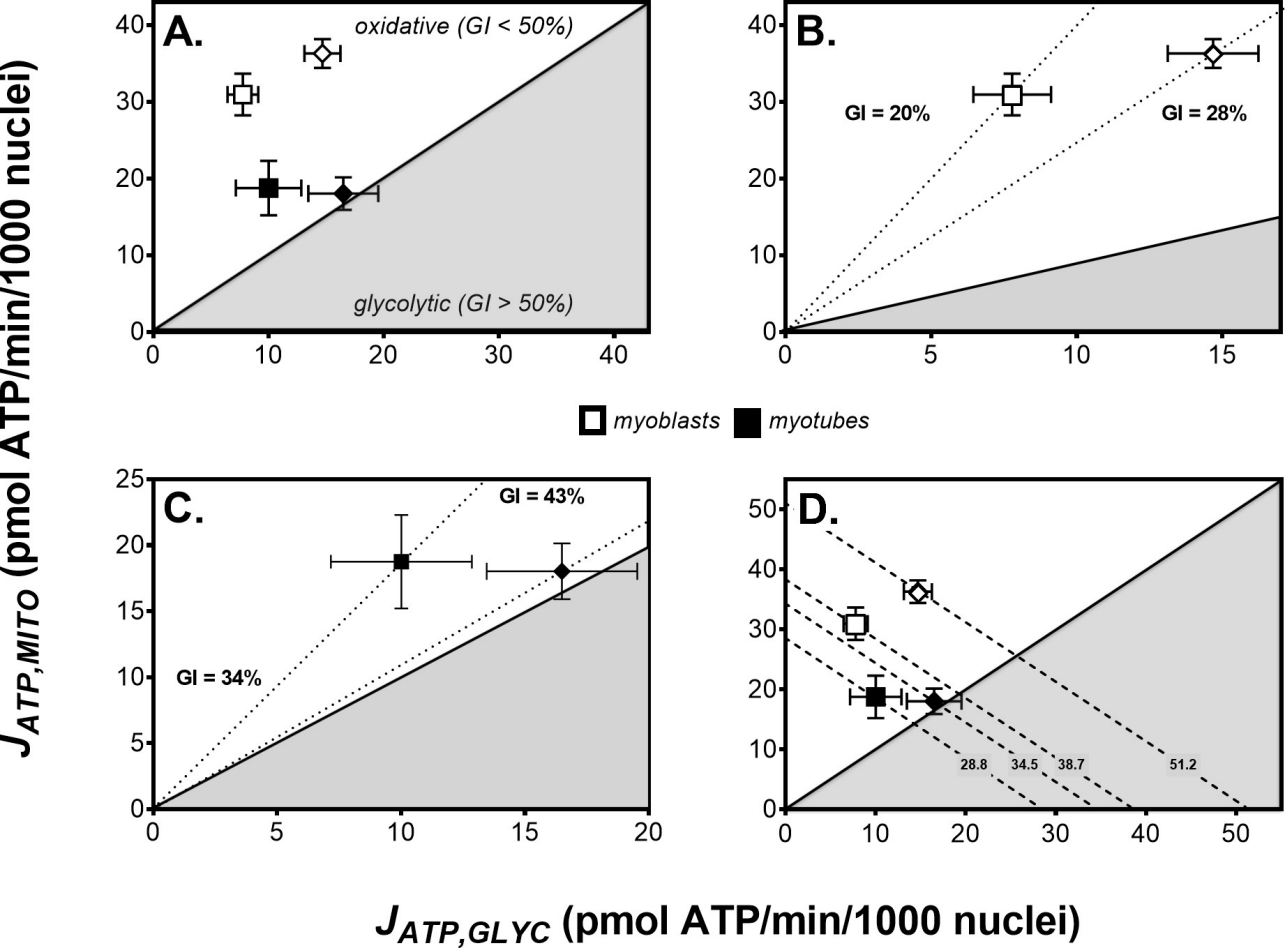
Nitrite increases the dependence of skeletal muscle cells on glycolytic ATP supply. Bioenergetic space plots [21] relate the rate of mitochondrial ATP supply (*J*_*ATP*,*MITO*_) to the rate of glycolytic ATP supply (*J*_*ATP*,*GLYC*_). Individual data obtained from experiments with static myoblasts and spontaneously contracting myotubes, in the absence (squares) or the presence (diamonds) of NaNO_2_ (0.75 and 5 μM for myoblasts and myotubes, respectively), were sourced from Fig 5. Data are means ± SEM of 12–19 well measurements sampled from 4–5 independent XF assays. Diagonal lines with positive slopes reflect the glycolytic index (GI), i.e., the percentage of total ATP synthesis from glycolysis. The white area above the GI_50%_ diagonal covers ‘oxidative’ bioenergetic space, whereas the grey shaded area below this diagonal covers the ‘glycolytic’ space. GI values indicated by the dotted lines in Panels **B** and **C** were calculated from the shown mean rates of oxidative and glycolytic ATP supply. The dashed diagonal lines with a slope of –1 in Panel **D** indicate space with the same total rate of ATP supply. The numbers labelling these lines are mean supply rates in pmol ATP/min/1000 nuclei.

The NaNO_2_ effect on myocellular ATP supply can be illustrated by ‘bioenergetic space’ plots that directly relate mitochondrial and glycolytic ATP synthesis rates [21]. Cells that make most of their ATP via oxidative metabolism occupy space above the main diagonal (from the origin with a slope of 1) of such plots (Fig 7A, clear area), and have a glycolytic index (GI) [21] that is lower than 50%. Cells that rely predominantly on glycolysis for their ATP supply occupy space below the diagonal (Fig 7A, shaded area) and have a GI higher than 50%. GI is defined as the percentage of total ATP supply from glycolysis (*cf*. Fig 5G and 5H) and is inversely related to the slope of diagonals that link the origin of the space plot to the position that reflects particular metabolic behaviour (Fig 7B and 7C, dotted lines).

Myoblasts and myotubes assayed with 5 mM glucose as sole metabolic fuel both have a GI below 50% (Fig 7A), and can thus be considered oxidative systems with most of their ATP made by mitochondria. Consistent with increased myocyte reliance on glycolytic ATP supply after differentiation (*cf*. Fig 2), the mean GI of myoblasts (Fig 7B– 20%) is lower than that of myotubes (Fig 7C– 34%). NaNO_2_ pushes the bioenergetic behaviour of both systems acutely towards a more glycolytic phenotype, raising the GI of myoblasts to 28% on average when applied at 0.75 μM (Fig 7B) and that of myotubes to 43% when applied at 5 μM (Fig 7C).

Diagonals in bioenergetic space plots with slopes of –1 link positions at which the rate of total ATP supply is identical, but is accounted for by differential relative contributions of glycolytic and oxidative ATP supply flux (Fig 7D, dashed lines). For instance, the total ATP supply is faster in myoblasts than in myotubes–even though the contractile differentiated L6 myocytes are more glycolytic (Fig 7C) than their non-differentiated static counterparts (Fig 7B)–and the respective steady states are thus described by separate negative diagonals. NaNO_2_-induced GI increments shift both steady states onto different diagonals (Fig 7D), which confirms NaNO_2_ has increased total ATP supply, and glycolytic ATP synthesis has not been raised by NaNO_2_ at the expense of oxidative phosphorylation. NaNO_2_ lifts the rate of total ATP synthesis from 38.7 to 51.2 pmol ATP/min/1000 nuclei when applied at 0.75 μM in myoblasts, and from 28.8 to 34.5 pmol ATP/min/1000 nuclei when applied at 5 μM in myotubes. This NaNO_2_ effect is statistically significant in myoblasts (*cf*. Fig 5E) and tends to significance in myotubes (*cf*. Fig 5F). Consistent with the data shown in Fig 2, the total ATP supply rates tend to be lower in myotubes than myoblasts, irrespective of variation between experiments.

### Nitrite does not affect glucose uptake

**Fig 8:**
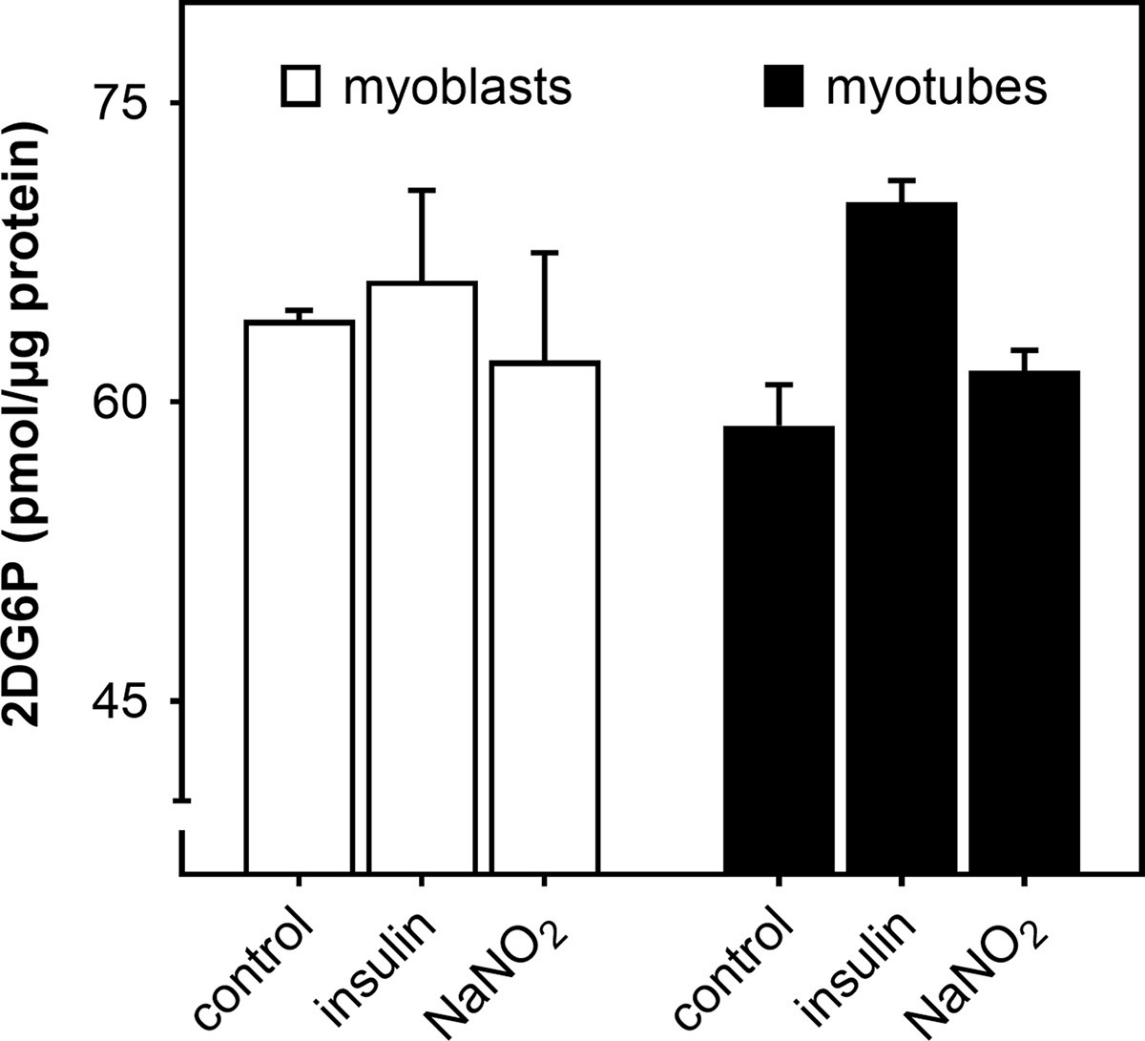
Nitrite does not affect glucose uptake by skeletal muscle cells. Static myoblasts (open bars) and spontaneously contracting myotubes (filled bars) were grown and assayed under the same conditions as those applied during extracellular flux analysis, and were exposed to 1 μM NaNO_2_ for 30 min with and without 100 nM human insulin. Glucose uptake was assayed as 2-deoxyglucose-6-phosphate (2DG6P) accumulated over a 30-min period. Data are means ± SEM of 3 independent experiments with each condition assayed in triplicate. Differences between differentiation state and assay conditions were evaluated by 2-way ANOVA and were found not significant.

When the bioenergetic behaviour of L6 myocytes is measured in buffer lacking metabolic fuel, injection of 2 mM glucose instantly stimulates acidification of the extracellular medium, but leaves oxygen consumption unaffected [22]. Such responses suggest the possibility that the NaNO_2_-induced increase in the glycolytic ATP synthesis rate is secondary to increased glucose availability. Indeed, nitrite has previously been reported to increase glucose uptake by cultured adipocytes [28], and we have seen acute NaNO_2_ stimulation of insulin-sensitive glucose uptake by L6 myocytes [29]. Because the regulation of cellular glucose uptake, for example by insulin, depends on the cells’ exact growth history and assay conditions [30], we measured accumulation of 2-deoxyglucose in myocytes cultured in the same way as those used for the bioenergetic studies, and applied assay conditions that were near-identical. Glucose uptake is unaffected by a ½-hour NaNO_2_ exposure in myocytes treated in this representative manner (Fig 8). The lack of glucose uptake phenotype holds for resting, insulin-unresponsive myoblasts as well as for spontaneously contracting, insulin-sensitive myotubes.

## Discussion

The extracellular flux data reported in this paper highlight how simultaneous measurement of oxygen consumption and medium acidification by cultured cells can offer valuable insight in cellular energy metabolism when such data are used to quantify ATP supply flux. We thus reveal that the rate of glycolytic ATP synthesis in skeletal muscle cells is increased by a ½-hour NaNO_2_ exposure. This acute stimulation of glycolytic ATP supply does not occur at the expense of mitochondrial ATP synthesis, which means NaNO_2_ raises the overall rate of ATP supply, and pushes the myocytes towards a more glycolytic bioenergetic phenotype. As the increase in the overall ATP synthesis rate emerges against a relatively stable oxygen uptake background, NaNO_2_ lowers the apparent oxygen cost of ATP supply. This effect is not clear from the ‘raw’ oxygen uptake and medium acidification data.

### Study limitations

The acute nitrite effects on the bioenergetics of cultured skeletal muscle cells are interesting in context of the reported benefit [12, 13] of dietary nitrate for human muscle performance, as nitrate increases the circulating nitrite concentration [5]. However, several limitations of our study’s design necessitate caution as to the physiological relevance of these *in vitro* findings. Firstly, we exposed our skeletal muscle cells to NaNO_2_ concentrations that are about 2 to 8 times higher in case of myoblasts and myotubes, respectively, than the 600 nM nitrite [5] to which human muscle is exposed after dietary nitrate supplementation. We thus emphasise that the some of the *in vitro* nitrite effects only become statistically significant at what may be considered supra-physiological levels. The concentration discrepancy is less than a single order of magnitude, however, and is perhaps off-set by an exposure time difference, as human muscle faces the increased nitrite levels considerably longer than the ½ h applied during the *in vitro* experiments. Secondly, like many experimental tissue culture models, we grew and assayed our myocytes under an ambient oxygen tension that will be much higher than the one that prevails in resting and contracting skeletal muscle [31, 32], and it may well be that known control of oxygen tension over muscle energy metabolism [33] influences the bioenergetic nitrite phenotype. Thirdly, we observe the nitrite effects both in static myoblasts and in contractile myotubes. We have not measured the relation between *in vitro* contraction and force generation, however, and it thus remains unclear to what extent spontaneous contractions mimic exercise workload. Fourthly, the physiological relevance of *rodent* myocytes is clearly debatable, but it is worth emphasis that L6 cells have proven a reliable model of skeletal muscle bioenergetics in our hands before, with behaviour that was highly reproducible in primary human myocytes [22, 29, 34]. In light of these study limitations, we do thus *not* claim that our data disclose how inorganic nitrate may lower the oxygen cost of exercise, but we suggest that the acute effects of nitrite are taken into account by future mechanistic models seeking to explain benefits of dietary nitrate for human muscle function.

### Oxygen cost of exercise

Dietary nitrate has been reported to lower the oxygen cost of human exercise by decreasing proton leak in skeletal muscle mitochondria, thus increasing coupling efficiency of oxidative phosphorylation [15]. Lowered proton leak was attributed to decreased protein expression of mitochondrial carriers whose contribution to proton leak is contentious, particularly under the bioenergetic conditions that prevail during exercise [14]. More recent human work suggests that lowered whole-body oxygen uptake may occur without mitochondrial efficiency changes [16, 17], a notion supported by research on various model systems [35–38]. Our data add to this body of corroborating support by showing that nitrite–whose circulating [5] and intra-myocellular [20] concentrations are raised after dietary nitrate exposure–does *not* increase coupling efficiency of oxidative phosphorylation in myoblasts (Fig 4A) or in spontaneously contracting myotubes (Fig 4C).

The oxygen cost of skeletal muscle work is not only set by coupling efficiency of oxidative phosphorylation, but also by the proportion of total ATP supply from glycolysis [21]. The data we report here demonstrate that nitrite acutely increases this proportion in cultured muscle cells, as is reflected by a rise in the myocellular glycolytic index (Fig 7). The raised GI follows from a nitrite-induced stimulation of glycolytic ATP supply, which occurs without loss of oxidative ATP supply. Mitochondrial oxygen uptake changes relatively little following nitrite exposure, whilst non-mitochondrial respiration decreases (Fig 3), which thus lowers the oxygen cost of total ATP synthesis (Fig 5). Notably, this apparent oxygen cost benefit does not reflect increased efficiency of oxidative metabolism, which remains unaffected (Fig 4A and 4C). The increased proportion of total ATP supply from glycolysis is reflected by nitrite-induced stimulation of lactate release from myotubes, as measured during extracellular flux assays. The elevated lactate release appears at odds with the reported lack of nitrate effect on the circulating lactate concentration [8], and with the increased endurance seen after dietary nitrate supplementation [39]. Notably, *in vivo* lactate kinetics are generally complex [40], which may underlie the variability of nitrate effects on the plasma lactate concentration. Indeed, improved muscle function after dietary nitrate supplementation does not always occur without lactate effect, but may also coincide with increased [41–43] and decreased [44] lactate levels. Limitations of *in vitro* experiments (see above) likely contribute to the lactate discrepancy.

Dietary nitrate effects on skeletal muscle function depend on fibre type composition [45]. For instance, nitrate increases oxygen perfusion [46] and calcium handling [47] of murine fast-twitch (type II) but not slow-switch (type I) fibres. Consistently, nitrate-lowering effects on the systemic oxygen uptake rate are most pronounced during exercise where type II fibres are recruited predominantly [48–50]. Nitrate selectivity is possibly related to the relatively low microvascular oxygen tension of type II fibres [51], as exercise benefits are more prominent in hypoxia [52, 53] than normoxia [54]. In this respect, it is worth emphasis that type II fibres are glycolytic systems [55] that perhaps benefit more than oxidative type I fibres [55] from the nitrite-induced stimulation of glycolytic ATP supply reported here (Fig 7).

### Mechanism of bioenergetic nitrite effect

Our results do not reveal the mechanism by which nitrite stimulates glycolytic ATP supply, but they exclude several possibilities. Changes in mitochondrial and glycolytic ATP synthesis are often reciprocal. For instance, an increased glycolytic ATP synthesis rate may arise as compensatory response to impeded mitochondrial ATP supply (Pasteur effect–*cf*. Fig 1A and 1B for opposite effects of oligomycin on respiration and proton production). Instead, an increase in glycolytic ATP supply may suppress oxidative ATP synthesis (Crabtree effect). However, it is unlikely that our nitrite phenotype reflects such ‘bioenergetic supply flexibility’ [21], as stimulation of glycolytic ATP supply does not coincide with lowered mitochondrial respiration. The maintained rate of oxidative phosphorylation (Fig 5) renders it unlikely that cytochrome *c* oxidase has been inhibited by nitrite-derived nitric oxide [56]. Dietary nitrate effects on glucose homeostasis [57] may be related to increased skeletal muscle glucose disposal [58]. Increased myocellular glucose availability could, in principle, explain the bioenergetic nitrite effect we report here (*cf*. [22]), but such an explanation is weakened by the lack of nitrite effect on 2-deoxyglucose uptake (Fig 8). Increased glucose availability could also be achieved by nitrite stimulation of glycogenolysis, a possibility that cannot be excluded by our data.

It is conceivable that glycolytic ATP supply is raised indirectly in response to increased ATP turnover as skeletal muscle ATP flux is predominantly demand-driven [59]. Since we see the glycolytic nitrite phenotype in spontaneously contracting myotubes and resting myoblasts, nitrite will have stimulated an ATP-consuming process that is common to both cell systems in this case, which would rule out stimulation of energy demand from increased contractile activity. If nitrite indeed stimulates ATP consumption, then it will also have to be explained why raised ATP demand is mostly met by glycolytic rather than mitochondrial ATP supply. Such an explanation likely relates to a difference in sensitivity of glycolytic and mitochondrial ATP supply to changes in the cell’s phosphorylation potential. In addition to, or instead of, indirect stimulation of glycolysis, it is of course also possible that the activity of one or more glycolytic enzymes is modulated directly by nitrite. Although the nitrite phenotype arises on a relatively short timescale, such modulation may involve upregulation of gene expression.

Irrespective of the site(s) of action, it also remains to be established whether the nitrite effect is direct or mediated by a nitrite-derivative. Whilst nitric oxide is held responsible for dietary nitrate protection against various pathologies [60], it remains uncertain if this radical species is involved in the human exercise phenotype [14, 61]. Indeed, the acute nitrite effect on the oxygen cost of ATP synthesis reported here is achieved at neutral pH and under an oxygen atmosphere (21%) that is hyperoxic from a physiological perspective, which disfavours nitrite reduction to nitric oxide.

### Concluding remarks

The quantitative analysis of myocellular bioenergetics reported here sheds fresh light on the elusive mechanism that underlies the exercise benefit of nitrate. The impact of our findings on human physiology remains to be fully elucidated, but we feel that the reported data will contribute towards the mechanistic understanding that will be crucial for achieving the full translational potential of dietary nitrate supplementation.

## Supporting information

S1 File Underlying data. This GraphPad Prism file contains data that underpin the figures presented in this paper.
(XLSX)
